# Shoulder Agony: A Painful Glenoid Cyst

**DOI:** 10.7759/cureus.33726

**Published:** 2023-01-12

**Authors:** Badrul Akmal Hisham Md Yusoff, Muhamad Karbela Reza Ramlan, Ahmad Farihan Mohd Don, Muhammad Ilyaas Muhammed Ali Noor, Norlelawati Mohamad

**Affiliations:** 1 Orthopaedic and Traumatology, Universiti Kebangsaan Malaysia, Kuala Lumpur, MYS; 2 Sports Medicine, Universiti Kebangsaan Malaysia, Kuala Lumpur, MYS

**Keywords:** pain, shoulder, cyst, ganglion, glenoid

## Abstract

A ganglion cyst is a benign cystic lesion that can occur intraosseously. It is commonly a multiloculated lesion that consists of fibrous tissue with mucoid change and is usually located in the subchondral bone adjacent to the joint. Here, we present a rare case of a 33-year-old woman who presented to our orthopedic clinic with a five-year history of shoulder pain that gradually worsened. The pain was worst after any activity involving the right shoulder, and it also occurred at night and disturbed her sleep. Physical examination showed that the patient had limitations on the extreme range of movement. MRI revealed a subchondral bone cyst at the glenoid fossa. The ganglion cyst was subsequently curetted and packed with a bone graft from the iliac bone. Postoperatively, the pain resolved and the patient’s range of movement improved. At the six-month follow-up, new bone formation over the void area caused by the cyst was observed on MRI.

## Introduction

A ganglion cyst is a benign cystic lesion that can occur intraosseously. It is commonly a multiloculated lesion that consists of fibrous tissue with mucoid change and is usually located in the subchondral bone adjacent to the joint [1]. The most common sites of intraosseous ganglia are at the hip, knee, and ankle [2]. Intraosseous ganglia of the glenoid are rare. Only 13 cases regarding intraosseous ganglia of the glenoid have been reported [2].

## Case presentation

A 33-year-old woman presented to our orthopedic clinic with a five-year history of right shoulder pain. The pain was localized to the right shoulder without any weakness, numbness, or associated neck pain. The pain was worse after any activity involving the use of the right shoulder. The patient also reported that the pain occurred at night and disturbed her sleep. She recalled an injury during a scuba diving session five years earlier when she dove into the sea in an awkward position, hitting the water with her right shoulder. The pain began at this point, but it was not severe enough for her to seek medical attention. The pain was affecting her more frequently and with worsening intensity in the year and a half prior to coming to our orthopedic clinic.

On physical examination, no obvious deformity, swelling, or erythema of the shoulder was observed. Active forward flexion of the right shoulder was from 0° to 130° and abduction was from 0° to 150°. The range of movement was limited because of pain and not weakness. All special tests for the shoulder yielded negative results.

**Figure 1:**
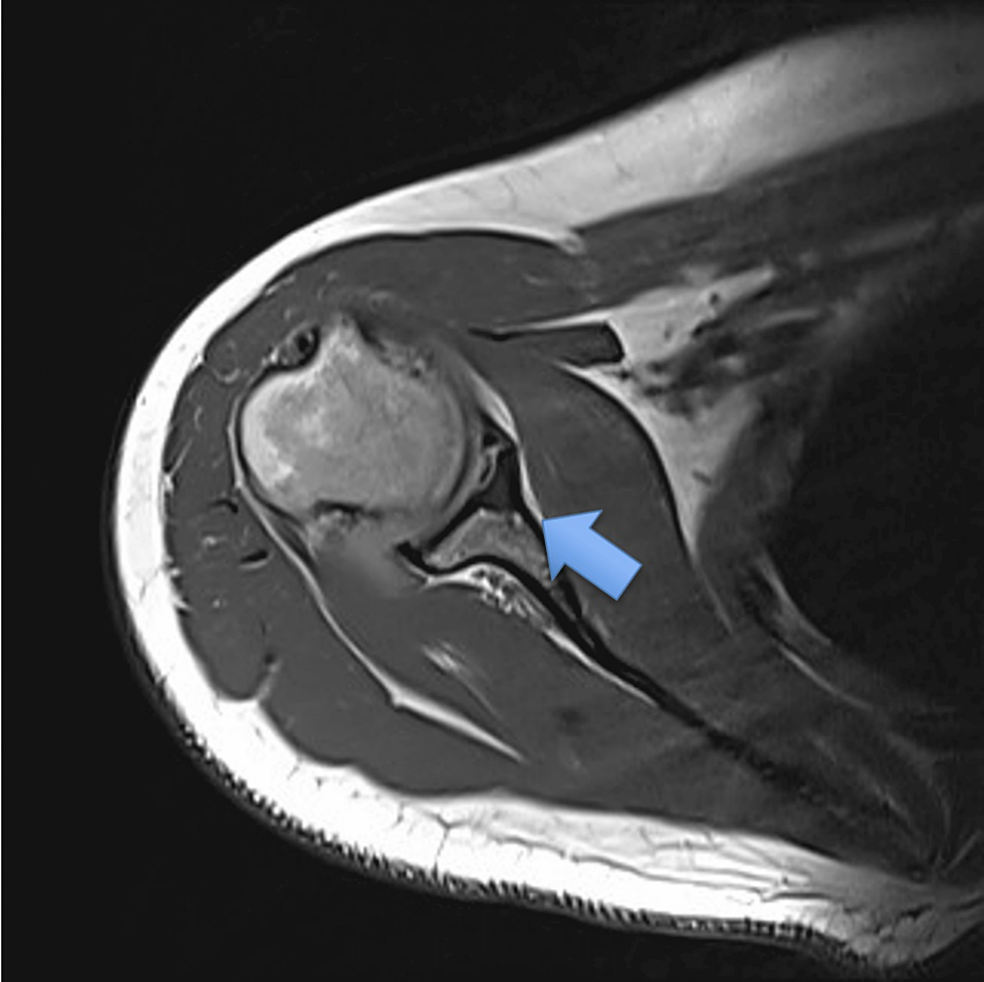
Axial view of MRI shows the glenoid cyst.

**Figure 2:**
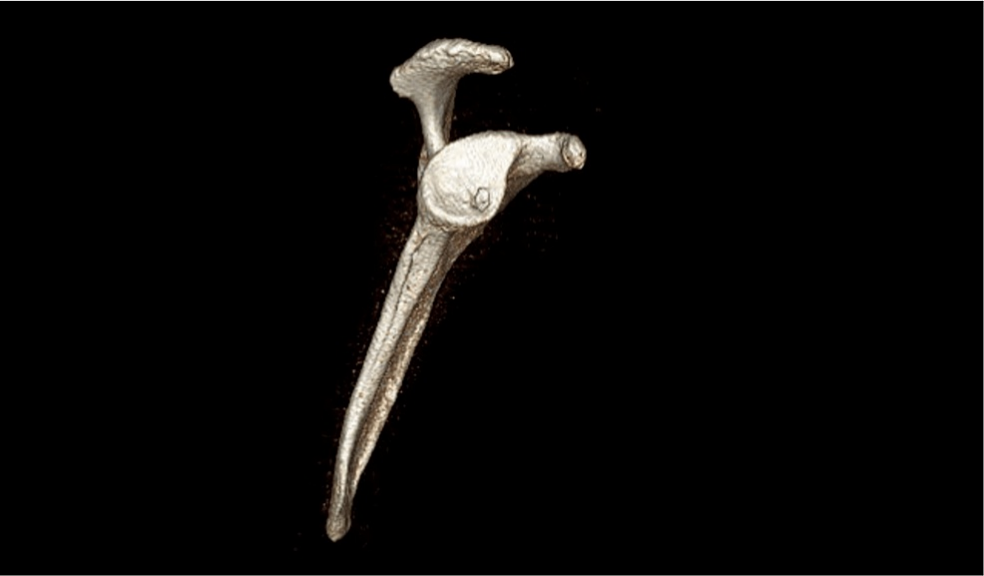
CT scan shows the location of the cyst.

A plain radiograph did not show any abnormalities, and MRI was subsequently ordered. MRI showed a subchondral bone cyst at the glenoid fossa (Figure 1). Preoperatively, a supplementary CT scan was also obtained (Figure 2).

**Figure 3:**
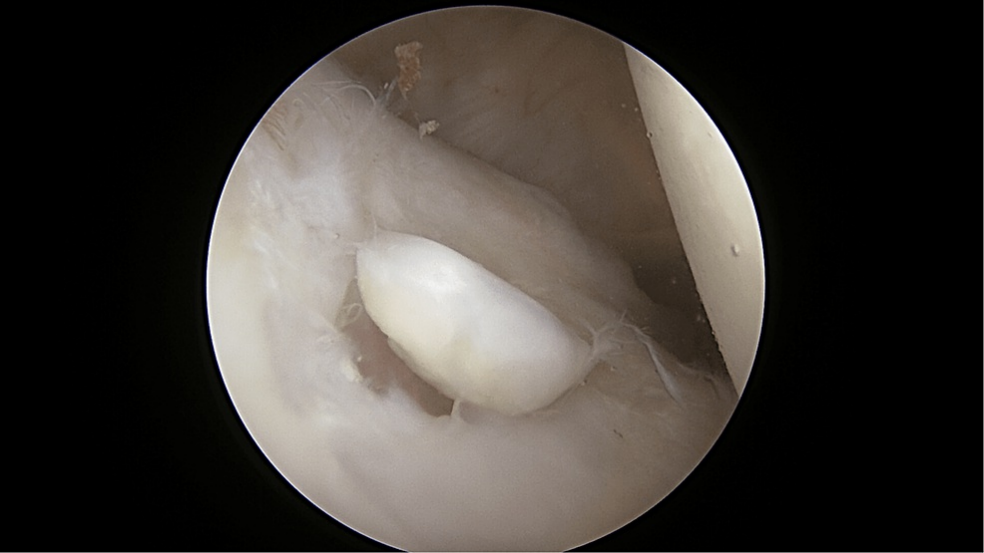
Loose body within the shoulder.

**Figure 4:**
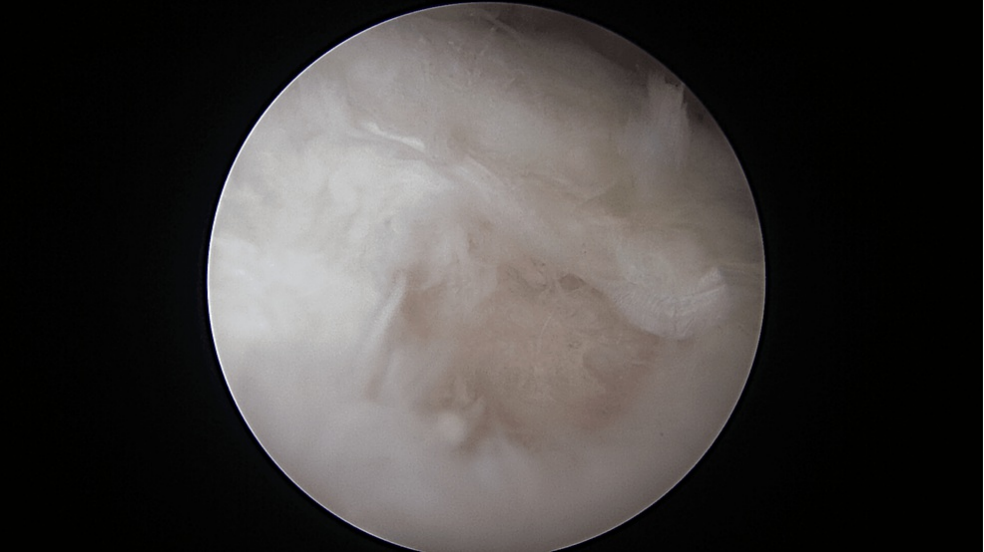
Defect area on the articular surface, with the cyst underneath it.

**Figure 5:**
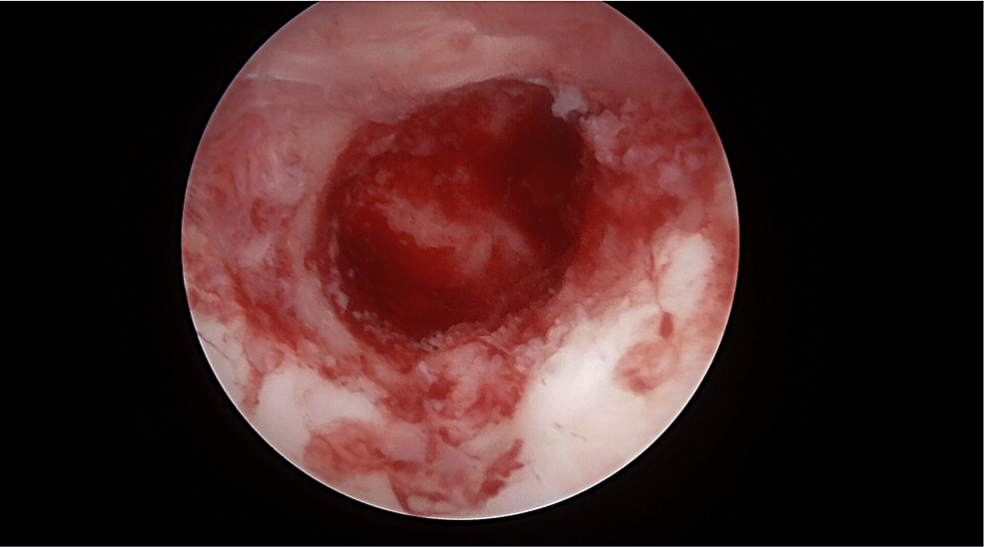
Post-curettage of the glenoid cyst.

**Figure 6:**
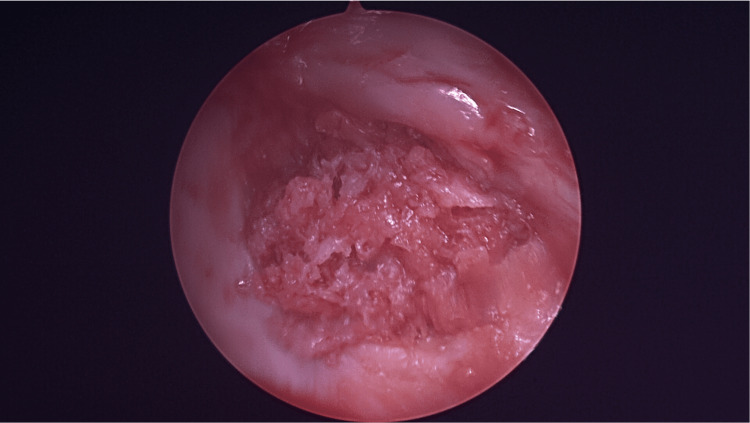
The defect area filled with autologous bone graft.

Diagnostic arthroscopy of the left shoulder was done through surgical exploration. The patient was placed in a beach chair position under general anesthesia. The surgery started with a diagnostic scope of her right shoulder. The diagnostic arthroscopy revealed a loose body measuring about 1.0 cm × 1.0 cm, which was identified as a piece of cartilage detached from the defect area (Figure 3), and a defect over the glenoid fossa at the lower anterior quadrant measuring 1.0 cm × 1.0 cm (Figure 4). The surgery was then converted to open surgery using the deltopectoral approach, and the cyst was curetted (Figure 5) and packed with an autologous bone graft (Figure 6) harvested from the left iliac crest. Postoperatively, the patient wore a slingshot shoulder brace and had complete immobilization of the right shoulder for two weeks. After two weeks, she was instructed to do a one-plane range of movement over the shoulder, which involved forward flexion and extension. Six weeks postoperatively, the patient was instructed to start doing full range of motion of the right shoulder. Immediately post-surgery, the patient reported that she did not have any resting pain or night pain.

Histopathology examination of the tissue sample revealed a combination of benign tissue composed of bone, cartilage, and fibrous tissue.

**Figure 7:**
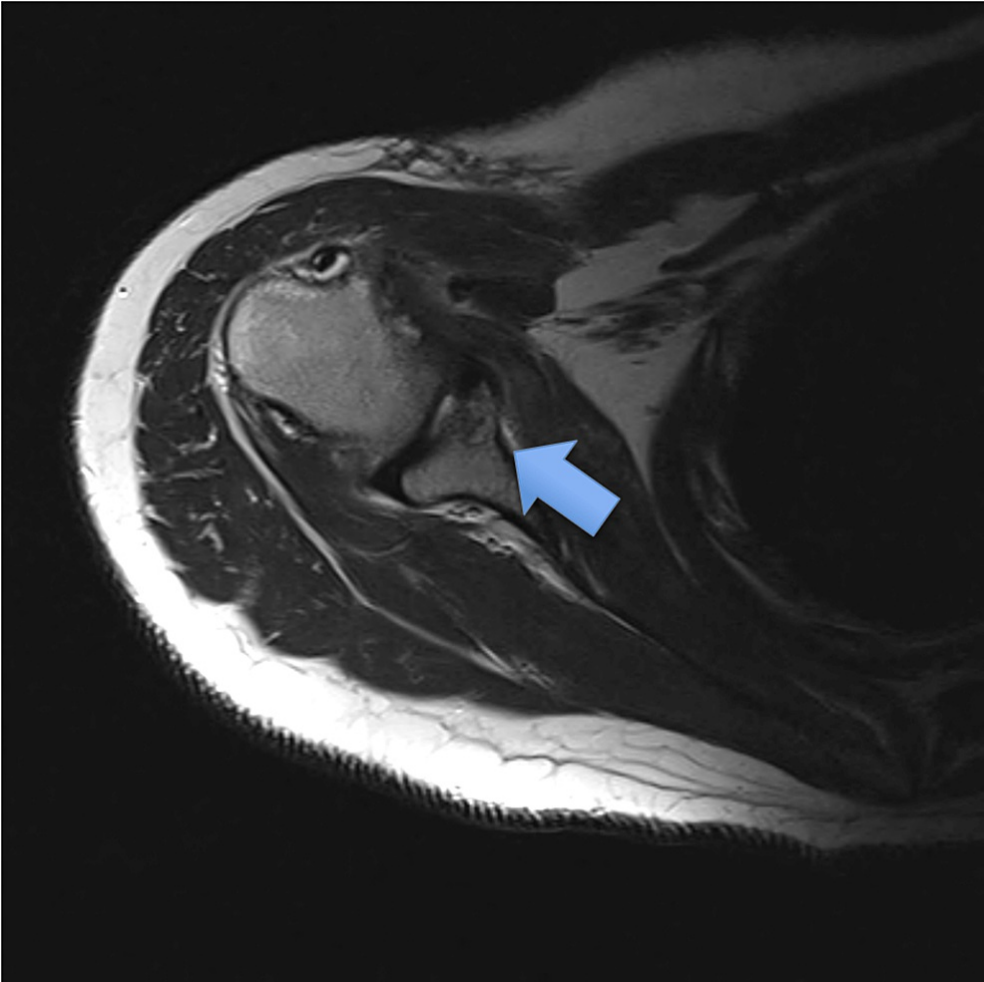
Callus formation over curretted glenoid cyst.

The patient was followed up regularly in our clinic postoperatively, and she has not experienced any pain since the surgery. She has a full range of movement for the shoulder after completing physiotherapy. An MRI of the shoulder was repeated six months postoperatively and showed that a callus had formed at the location of the cyst (Figure 7).

## Discussion

Intraosseous ganglia can be classified into two categories, namely, type 1 when they are isolated, and type 2 when the ganglia invade the adjacent soft tissue [2,3]. These lesions rarely occur in the glenoid [2,4], and when a glenoid ganglion does occur, the most common location is at the inferior pole [4]. If the cyst expands, it can cause an osteoarticular fracture with the destruction of subchondral bone [5]. If the cyst expands to the spinoglenoid notch, it can cause entrapment of the suprascapular nerve [5,6]. A common symptom is pain over the posterolateral area of the shoulder, which also occurs when the patient tries to sleep on the affected side [6].

Intraosseous ganglia typically lack evidence of trauma, including dislocation. Pain is usually around the shoulder, with or without a limited range of movement. On physical examination, all tests specific for rotator cuff dysfunction are negative. A plain radiograph can be helpful in cases in which a ganglion is large but less helpful with a smaller lesion. A CT scan enables seeing intraosseous ganglia and offers better visualization than a plain radiograph. An MRI scan can also be used in identifying the intrinsic structure of the ganglia [4].

Surgical decompression of the ganglia is required to relieve the symptoms [2,4-6]. The surgery can be done via an open or arthroscopic approach. The arthroscopic method is not suitable if the ganglia are multiloculated. Decompression can be done by curettage of the ganglia, followed by packing the defect area with a bone graft.

## Conclusions

In a case of a painful shoulder, especially when the pain occurs at night in a young patient, we suggest including a subchondral glenoid cyst in the differential diagnosis. For the treatment, we suggest the removal of the cyst, followed by bone graft packing of the void area. This management successfully treated our patient’s pain and improved her quality of life.

## References

[1] Schajowicz F (1972). Histological Typing of Bone Tumours.

[2] Tudisco C, Bisicchia S (2011). Intraosseous ganglion with impending fracture of the glenoid. Orthopedics.

[3] Schajowicz F, Clavel Sainz M, Slullitel JA (1979). Juxta-articular bone cysts (intra-osseous ganglia): a clinicopathological study of eighty-eight cases. J Bone Joint Surg Br.

[4] Kligman M, Roffman M (2000). Intraosseous ganglia of glenoid. J South Orthop Assoc.

[5] Kim JR, Wang SI (2017). Suprascapular nerve entrapment caused by an intraosseous ganglion of the scapula: A case report. Medicine (Baltimore).

[6] Ishimaru D, Nagano A, Terabayashi N, Nishimoto Y, Akiyama H (2017). Suprascapular nerve entrapment caused by protrusion of an intraosseous ganglion of the glenoid into the spinoglenoid notch: a rare cause of posterior shoulder pain. Case Rep Orthop.

